# Ventricular fibrillation arrest with cardiomyopathy in the setting of exogenous T3 consumption in a previously healthy young male

**DOI:** 10.1016/j.jccase.2025.01.002

**Published:** 2025-01-30

**Authors:** Tobin Mathew, Kevin S. Tang, Fahad Gul, Sareen Sandhu, Omid Vadpey, Qin Yang, David Donaldson

**Affiliations:** a University of California, Irvine Medical Center, Department of Medicine, Division of Cardiology, Orange, CA, USA; b University of California, Irvine Medical Center, Department of Medicine, Division of Endocrinology, Orange, CA, USA

**Keywords:** Ventricular fibrillation, Sudden cardiac death, Exogenous hyperthyroidism, Non-ischemic cardiomyopathy

## Abstract

Ventricular fibrillation (VF) is an often-fatal cardiac arrhythmia with increased prevalence in those with structural heart disease, congestive heart failure, and history of myocardial infarction. Our case describes a young adult male who presented with VF arrest and new onset cardiomyopathy in the setting of exogenous testosterone and triiodothyronine supplementation. Comprehensive work-up demonstrated a severely reduced ejection fraction, no angiographically significant coronary artery disease on invasive coronary angiography, and evidence of right ventricular mid-lateral wall scarring on electrophysiology study and cardiac magnetic resonance imaging. Exogenous thyroid hormone and testosterone supplementation have been independently associated with development of dilated cardiomyopathy; however, VF arrest has rarely been described in otherwise previously healthy individuals with concomitant use of these substances. Optimal management, risk stratification, and prognosis in this population remains unknown. Our case identifies an at-risk population of sudden cardiac death where appropriate work-up and shared clinical decision-making is essential to improved patient outcomes and quality of life.

**Learning objective:**

Exogenous triiodothyronine (T3) intake may be a risk factor for the development of acute cardiomyopathy, as cardiac myocytes directly uptake T3 which can induce arrhythmias and cardiac arrest. This process may be separate from tachycardia-mediated cardiomyopathy. Prognosis of hyperthyroid induced ventricular fibrillation and cardiomyopathy is unclear. Reversing the hyperthyroid state may reduce the risk of repeat sudden cardiac death. The decision for secondary prevention should be a joint decision understanding patient-specific risk factors and goals.

## Highlights

• Exogenous triiodothyronine intake may be a risk factor for the development of acute cardiomyopathy.
• Cardiac myocytes will directly uptake T3 which can induce arrhythmias and cardiomyopathy
• Prognosis of hyperthyroid induced ventricular fibrillation and cardiomyopathy is unclear.
• Reversing the hyperthyroid state may reduce the risk of repeat SCD.

## Introduction

Ventricular fibrillation (VF) is a cause of sudden cardiac death (SCD) and associated with patients with underlying cardiac disease including structural heart disease, congestive heart failure (CHF), prior myocardial infarction, accessory electrical conduction pathways, and electrolyte and endocrine abnormalities [1]. Thyroid dysfunction is independently associated with myriad adverse cardiovascular outcomes. Hyperthyroidism leading to VF arrest is not well described, but there are case reports of such events in the context of severe thyroid dysfunction [2]. Use of exogenous hormone supplementation is also associated with cardiac abnormalities, but current literature is sparse on diagnostic and management protocols for SCD and non-ischemic cardiomyopathy in this setting. Our case highlights the challenges in prognostication for a previously healthy young patient who survived sudden cardiac arrest.

## Case report

A 25-year-old male with no past medical history presented after collapsing in a theme park and was found to be in VF arrest. Cardiopulmonary resuscitation was administered immediately; he was defibrillated twice in the field with subsequent return of spontaneous circulation and baseline mentation. Although not available, the rhythm strips were reviewed by emergency medicine physicians and cardiologists and confirmed VF arrest. On arrival to the emergency room, initial physical examination revealed an alert and responsive adult male found to be afebrile, normotensive (101/49 mmHg), tachycardic with a regular rhythm and rate in the 130 s, and hypoxic to 75 % on room air.

He reported fatigue without chest pain, dyspnea, palpitations, syncope, or lightheadedness prior to the incident. Family history was negative for any cardiovascular disease or SCD. Review of systems revealed a bandlike headache for two days along with bilateral hand tremor and diaphoresis. The patient had been a bodybuilder for nine years and used performance-enhancing drugs, including stanozolol and clenbuterol. He reported taking one 200-μg pill of triiodothyronine (T3) two weeks prior to presentation, but history obtained from family members suggested more significant consumption.

**Fig. 1:**
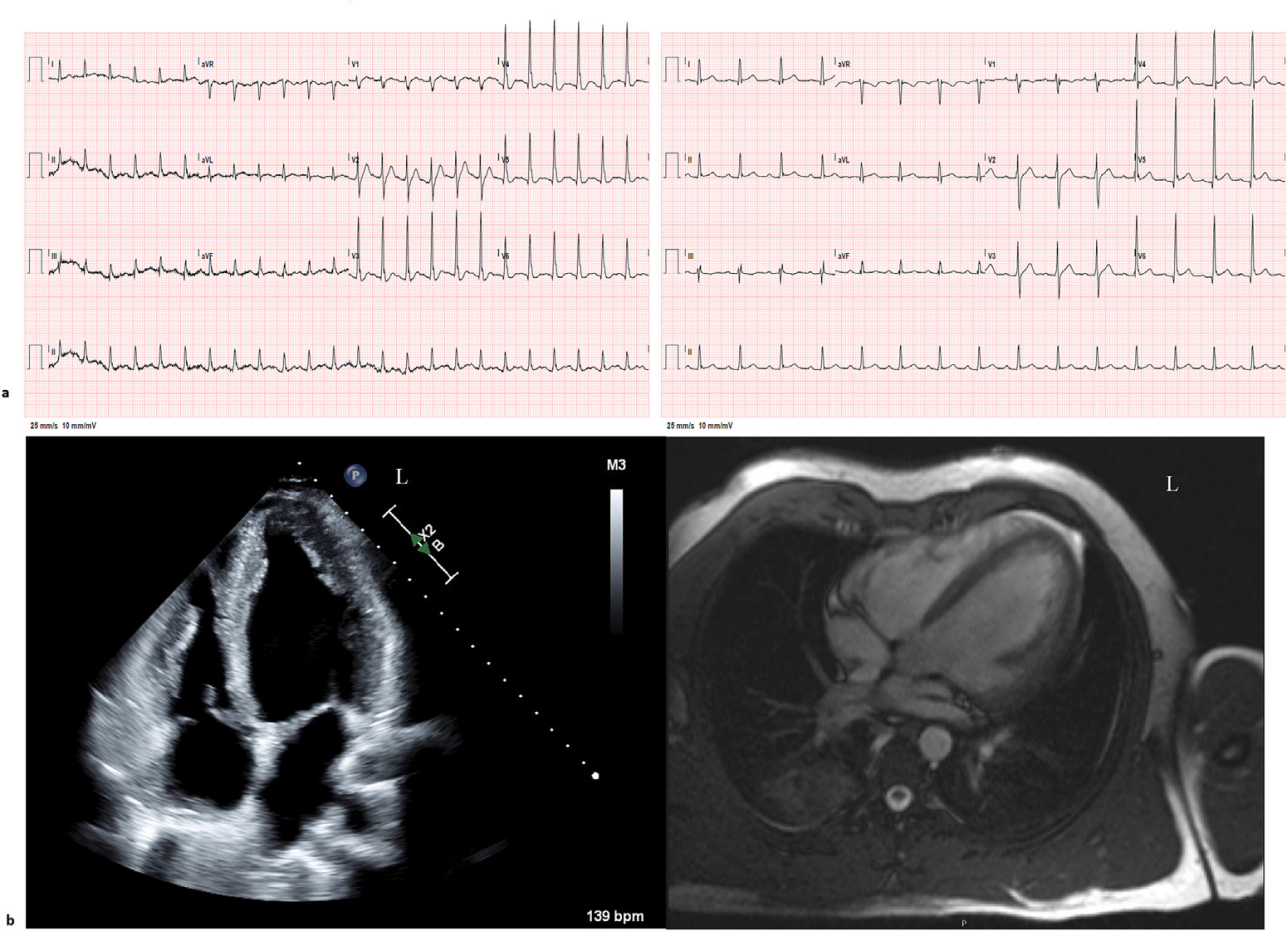
(a) Electrocardiogram on admission (left) demonstrating sinus tachycardia and two days after admission (right) demonstrating normal sinus rhythm. (b) TTE (left) in apical-4 chamber view and cardiac MRI with contrast (right), both pictured at end-diastole of the cardiac cycle. The LV is dilated in both studies. The cardiac MRI does not demonstrate late gadolinium enhancement. TTE: LV-estimated EF 30 % by biplane without regional wall motion abnormalities, end-diastolic internal diameter 5.63 cm. MRI: Severe global hypokinesis of the LV walls with estimated EF 40 %, normalized end-diastolic volume 90 mL/m^2^, normalized end-systolic volume 54 mL/m^2^, normalized stroke volume 36 mL/m^2^, normalized cardiac index 2.7 L/min/m^2^. EF, ejection fraction; LV, left ventricular; MRI, magnetic resonance imaging; TTE, transthoracic echocardiography.

Initial laboratory work-up was significant for thyroid stimulating hormone of 0.016 μIU/mL, free thyroxine of 0.45 ng/dL, and elevated total T3 of 501 ng/dL (Table 1). Urine comprehensive drug screen was negative. Computed tomography of the head and chest with contrast were unremarkable. Electrocardiogram (ECG) demonstrated sinus tachycardia with a rate of 145 and transthoracic echocardiogram (TTE) was notable for global cardiomyopathy with severely decreased left ventricular (LV) systolic function with ejection fraction (EF) 30 % without regional wall motion abnormalities and decreased right ventricular (RV) systolic function (Fig. 1). The patient was started on amiodarone for VF and propranolol. Thyroid ultrasound was unremarkable. Endocrinological analysis affirmed the hyperthyroidism was secondary to exogenous T3 intake. Transaminitis resolved by day two and high-sensitivity-troponin peaked at 1937 ng/L. T3 nadired at 42 ng/dL, and the patient was started on levothyroxine supplementation for expected rebound hypothyroidism.

**Table 1:** The patient's laboratory data during inpatient admission.

Laboratory test	Patient value	Reference range
Sodium (mmol/L)	140	136–145
Potassium (mmol/L)	4.5	3.5–5.1
Chloride (mmol/L)	101	98–107
Bicarbonate (mmol/L)	**20**	21–31
Blood urea nitrogen (mg/dL)	12	7–25
Creatinine (mg/dL)	1.2	0.7–1.3
Glucose (mg/dL)	**130**	70–115
Magnesium (mg/dL)	2.0	1.9–2.7
Phosphorus (mg/dL)	**10.2**	2.5–5.0
Creatine phosphokinase (U/L)	**840**	30–223
White blood cell (K/μL)	9.3	4.0–10.5
Hemoglobin (g/dL)	15.4	13.5–16.9
Platelet (K/μL)	289	150–400
Calcium (mg/dL)	9.1	8.6–10.3
Total Protein (g/dL)	6.3	6–8.3
Albumin (g/dL)	**3.9**	4.2–5.5
AST (IU/L)	**331**	13–39
ALT (IU/L)	**353**	7–52
Alkaline phosphatase (IU/L)	58	34–104
Total bilirubin (mg/dL)	1.2	0.0–1.4
Thyroid stimulating hormone (μU/mL)	**0.016**	0.450–4.120
Free T4 (ng/dL)	**0.45**	0.60–1.12
INR	1.04	0.89–1.11
Lactic acid (mmol/L)	**9.6**	0.5–2.0
hs Troponin (ng/L)	**39**	0–20
hs Troponin (4-hour mark)	**937**	0–20
hs Troponin (9-hour mark)	**1937**	0–20
Brain natriuretic peptide (pg/mL)	12	0–100
Low-density lipoprotein (mg/dL)	32	<200
High-density lipoprotein (mg/dL)	**21**	>40
Total cholesterol (mg/dL)	67	<200
Total T3 (ng/dL)	**501**	80–210
Thyroglobulin (ng/mL)	5.5	1.6–50
Thyroglobulin antibody (IU/mL)	<1	0–4
Thyroid peroxidase antibody (IU/mL)	<1	0–9
Thyroid stimulating antibody (TSI index)	<1.0	≤1.3
C-reactive protein (mg/dL)	0.4	0.0–1.0
Cortisol (random) (μg/dL)	24 (day 1) 24 (day 2)	AM: 6–28, PM: 3–16
Adrenocorticotropic hormone (pg/mL)	**292**	0–45
Growth hormone (ng/mL)	0.5	0–1
Insulin growth factor-1 (ng/mL)	99	99–283
Testosterone (pg/mL)	129	49–190

Abnormal values are shown in bold.

AST, aspartate transaminase; ALT, alanine transaminase; T4, thyroxine; T3, triiodothyronine; INR, international normalized ratio; hs-troponin, high-sensitivity troponin.

The patient began extensive cardiology work-up including coronary angiography, showing non-obstructed coronary arteries. Cardiac magnetic resonance imaging (MRI) (Fig. 1b) revealed mild RV enlargement with hypokinesis, without delays. Subsequently, an electrophysiology study (EPS) was performed, described in the Online Methods, which demonstrated rare premature ventricular complexes (PVCs) but no inducible arrhythmias or J waves.

Despite the negative work-up, nonreversible causes of non-ischemic cardiomyopathy could not be definitively ruled out. The diagnostic and prognostic challenges were discussed with the patient and his family, and a joint decision was made to pursue implantable subcutaneous cardioverter defibrillator (S-ICD) implantation (EMBLEM™ MRI S-ICD System; Boston Scientific Corp., Marlborough, MA, USA); it was inserted without incident. He was discharged with oral amiodarone, levothyroxine, losartan, and metoprolol succinate, and scheduled for follow-up with regard to heart failure, electrophysiology, and endocrinology, and was referred for comprehensive genetic testing. The patient has since followed up for post-device interrogation which did not demonstrate further arrhythmias or shocks. Resolution of his underlying cardiomyopathy on appropriate guideline-directed medical therapy is under way.

## Discussion

**Fig. 2:**
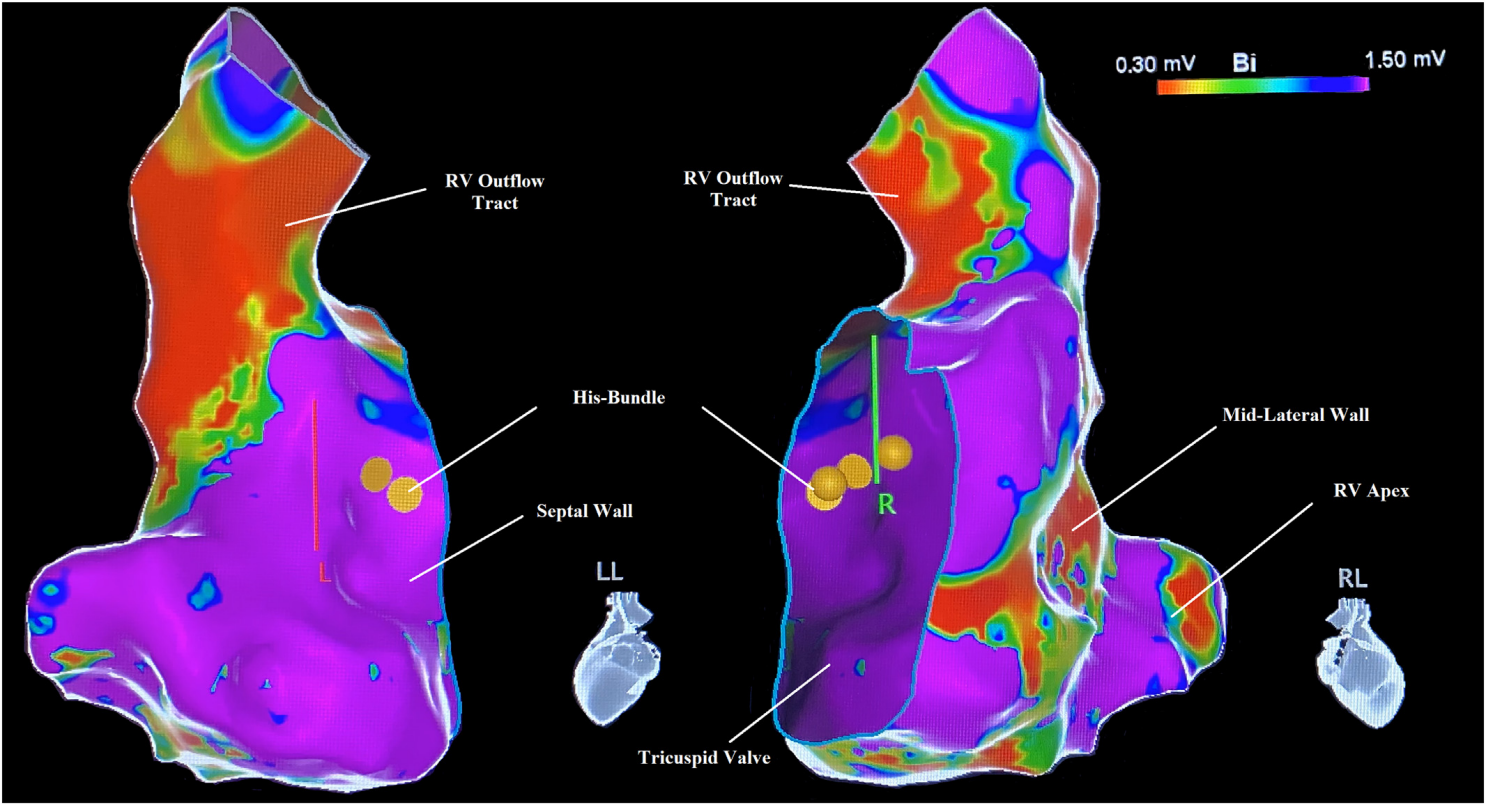
Electrophysiology study cardiac mapping of the RV in the left lateral (LL) view (left) and right lateral (RL) view (right). Legend depicts color spectrum indicating tissue voltage, with red indicating poor conductivity and purple indicating normal conductivity. Small-moderate sized band of poor conduction is seen at the mid-lateral wall, suggestive of scarring. RV outflow tract shows low voltage, which is normal. RV, right ventricular.

The presented patient is a young adult male with a history of exogenous intake of T3 presenting with SCD from VF. A variety of cardiac diseases are associated with VF, including structural heart disease, conduction abnormalities, and prolonged QT syndromes. Our patient presented with several abnormalities that likely predisposed him to VF, including new dilated cardiomyopathy, thyrotoxicosis, and small area of RV free wall scarring seen on endocardial mapping (Fig. 2).

The patient underwent a sequence of testing to rule out common causes of VF, cardiomyopathy, and CHF. TTE revealed globally reduced biventricular function. Coronary angiogram demonstrated no significant coronary disease, and cardiac MRI was not suggestive of restrictive or infiltrative etiology. No QT abnormalities were observed, and there was no family history of arrhythmia or structural cardiomyopathy. The EPS showed minimal scar in the RV free wall and infrequent PVCs but was negative for significant arrhythmogenic focus. It is unclear if RV outflow tract endocardial low voltage is associated with VF occurrence, although there is literature to suggest this mechanism in patients with Brugada syndrome [3]. RV outflow tract scarring may not correlate with electrical abnormalities but has a potential as an arrhythmogenic focus. There are theorized correlations with idiopathic PVC origin and early arrhythmogenic RV cardiomyopathy [4]. However, the EPS did not induce any such arrythmias and his inpatient stay was without catecholaminergic polymorphic ventricular tachycardia nor clear epsilon waves on serial ECG or telemetry monitoring, making arrhythmogenic RV dysplasia unlikely.

The patient's thyroid dysfunction was his distinguishing feature. New VF or other ventricular tachyarrhythmias are independently associated with hyperthyroidism [5]. However, his presentation was unique as he developed VF cardiac arrest in the absence of other symptoms of thyrotoxicosis. Dysregulation of thyroid hormone can lead to cardiovascular comorbidities including arrhythmias and CHF, and testing and treatment for underlying thyroid dysfunction is recommended in the setting of cardiac disease [6]. T3 is the predominant form acting on cardiomyocytes, binding to thyroid hormone receptors and leading to increased translation of β-1 adrenergic receptors, enhancing sensitivity to catecholamines which decreases VF threshold [6]. This interaction leads to tachycardia and increased myocardial contractility. There is a well-established link between systolic CHF and arrhythmia (classically atrial fibrillation) with thyroid dysfunction, but there is scant literature on VF. In our patient, it is difficult to ascertain the cause of the arrhythmia from the direct effect of T3 versus cardiomyopathy. Of note, the patient's adrenocorticotropic hormone level was elevated, but random cortisol levels measured at separate occasions were normal, which rules out a confounding steroidal effect.

Elevated cardiac output from hyperthyroidism can lead to high-output CHF due to catecholamine response followed by worsening ventricular function in later stages [7]. Despite reduced biventricular function of unknown duration, our patient presented in the absence of clinical signs of CHF. Given the short course of supplemental thyroid hormone, hyperthyroid-induced tachycardia-mediated-cardiomyopathy is less likely; 6 % of patients with thyrotoxicosis develop CHF symptoms with <1 % developing reduced ejection fraction; however, thyrotoxicosis has been found to also be associated with reduced RV systolic function, which was seen in our patient [8]. Other possibilities include spontaneous coronary artery spasm or testosterone hormone-mediated effects on cardiac repolarization causing ventricular arrhythmia. Hyperthyroidism has rarely been described to cause coronary artery spasm which may cause a range of presentations from mild palpitations to an acute coronary syndrome [9]. Given the rarity and lack of symptoms, we deemed this to be less likely. Exogenous testosterone supplementation has also been suggested to increase risk for a Brugada-like phenomenon, with the effects of testosterone hormone on modulating L-type calcium channels and cardiac repolarization [10]. While our patient did also endorse a history of testosterone use, his ECG was unrevealing for a Brugada-like phenotype, and he was not taking adrenergic supplementation at that time.

Our patient's prognosis is unclear given the unknown dynamic of various potential etiologies. If his non-ischemic cardiomyopathy developed from a reversible cause (e.g. hyperthyroidism), there may be recovery of cardiac function and low risk of future events. Extensive discussion was held on the risks and benefits of ICD implantation versus close monitoring on guideline-directed medical treatment. In patients presenting with clear reversible causes of VF, it may be reasonable to consider a wearable cardioverter defibrillator for secondary prevention while reversible causes are addressed. There is no definitive evidence that the patient's exogenous thyroid consumption directly led to his VF or cardiomyopathy. If the patient has an undiagnosed accessory pathway that was stimulated by the hypermetabolic state, he may have a persistently elevated risk of SCD. Without knowing the reversibility of his cardiomyopathy, the decision was to implant an ICD for secondary prevention. Meanwhile, the patient was referred for comprehensive genetic testing, including for conditions such as arrhythmogenic RV cardiomyopathy, the results of which are still pending.

## Conclusions

This case describes a unique presentation of thyrotoxicosis with VF arrest and the development of a non-ischemic cardiomyopathy, which may be secondary to exogenous T3-induced hyperthyroidism. Many weight-loss and muscle-building supplements are known to affect cardiac structure and function, which portends a theoretical risk of electrical abnormalities including VF. The prognosis of this case remains unclear; if the underlying condition is addressed and hormonal homeostasis is achieved, it is possible for a positive prognosis without recurrence. However, in the setting of uncertain VF etiology, it remains important to have a comprehensive risk-benefit discussion on ICD implantation for definitive secondary prevention.

## Consent

The patient involved in this case report has given their informed consent for the case report to be published.

## Declaration of competing interest

The authors declare no conflict of interest to disclose.

## Appendix A Supplementary data

Supplementary material Image 1
