# Supplementary material for: Ventricular fibrillation arrest with cardiomyopathy in the setting of exogenous T3 consumption in a previously healthy young male
Source: J Cardiol Cases. 2025 Jan 30;31(4):113–6. doi: 10.1016/j.jccase.2025.01.002 (PMC12130823; doi:10.1016/j.jccase.2025.01.002)
Supplement: Supplementary file 1 — Supplementary material [file mmc1.docx]

**Online methods**

Electrophysiology study (EPS) with CARTO 3-D (Biosense Webster, Diamond Bar, CA, USA) mapping system (standard atrial and ventricular protocols, with and without isoproterenol) was performed. Three catheters were inserted, including a single-decapolar catheter into the coronary sinus (CS), a quad catheter into the high-right-atrium (HRa) and one in right ventricular (RV) apex (RVa). With direct pacing from the HRa and RVa with and without 3 µg of isoproterenol, there was no evidence of a bypass tract and no dual atrioventricular nodal (AVN) physiology. The CS catheter was replaced with a His-bundle mapping catheter with baseline HRa to His interval of 125 milliseconds (ms), His to QRS of 40 ms, and PR 178 ms, QRS 877 ms, PP 1002 ms, and QT 401 ms. Baseline AVN effective refractory period was 600-510 ms; with isoproterenol, it was 600/410 ms. Endocardial mapping was performed with a PentaRay catheter of the RV, His bundle, and RV outflow tract, demonstrating a small size band of low potential electrograms <0.5 mV localized at the basal segment of the RV lateral wall (Fig. 2). A comprehensive EPS was performed at the RVa and RV outflow tract, using UPENN protocol with a drive train of 600 ms and 400 ms with a drive train of 8 beats and extra beats with a lower limit of 200 ms to the ventricular effective refractory period.
